# Extracorporeal Photopheresis—An Overview

**DOI:** 10.3389/fmed.2018.00236

**Published:** 2018-08-27

**Authors:** Ara Cho, Christian Jantschitsch, Robert Knobler

**Affiliations:** Department of Dermatology, Medizinische Universität Wien, Vienna, Austria

**Keywords:** ECP, ultraviolet A, CTCL, GVHD, scleroderma, solid organ transplantation

## Abstract

Extracorporeal photopheresis (ECP) has been in clinical use for over three decades after receiving FDA approval for the palliative treatment of the Sézary Syndrome variant of cutaneous T-cell lymphoma (CTCL) in 1988. After the first positive experiences with CTCL, additional indications have been successfully explored including areas such as graft-vs.-host disease (GVHD), scleroderma, and solid organ transplantation. The mechanism of action is still not fully resolved, but important steps in understanding ECP in recent years have been very informative. Originally, the primary hypothesis stated that psoralen and ultraviolet A (UVA) in combination induce apoptosis in the treated immune cells. This view shifted in favor of dendritic cell initiation, modification of the cytokine profile and stimulation of several T-cell lineages, in particular regulatory T-cells. A number of ECP guidelines have been produced to optimize treatment regimens in the clinical context. In CTCL, enough evidence is available for the use of ECP as a first line treatment for Sézary Syndrome (SS), but also as a second line or rescue treatment in therapy-refractory forms of mycosis fungoides (MF). ECP in the treatment of acute and chronic GVHD has shown promising results as second line therapy in steroid-refractory presentations. In solid organ transplantation, ECP has been used to increase tissue tolerance and decrease infections with opportunistic pathogens, attributed to the use of high doses of immunosuppressive medication. Infection with cytomegalovirus (CMV) remains a limiting factor affecting survival in solid organ transplantation and the role of ECP will be discussed in this review. A trend toward prophylactic use of ECP can be observed and may further contribute to improve the outcome in many patients. To further deepen our knowledge of ECP and thus facilitate its use in patients that potentially benefit most from it, future prospective randomized trials are urgently needed in this rapidly growing field. The aim of this review is to (1) introduce the method, (2) give an overview where ECP has shown promising effects and has become an essential part of treatment protocols, and (3) to give recommendations on how to proceed in numerous indications.

## Introduction

**Figure 1:**
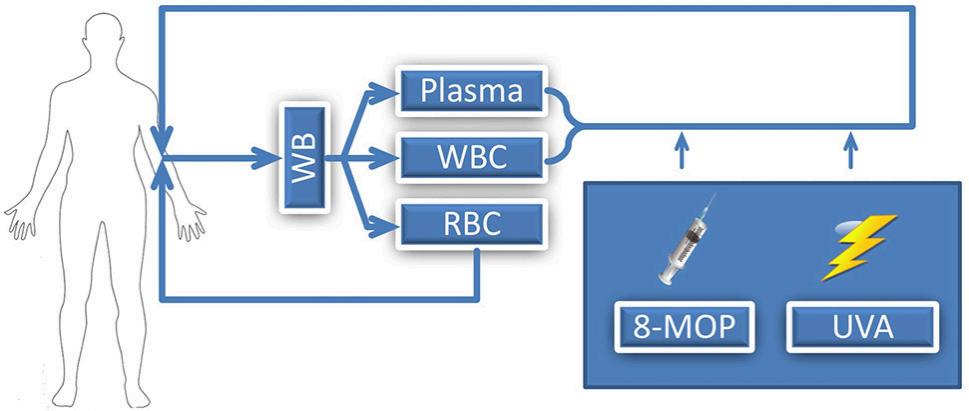
Illustration of ECP procedure; WB, whole blood; WBC, white blood cells; RBC, red blood cells; 8-MOP, 8-methoxypsoralen; UVA, Ultraviolet A.

Extracorporeal photopheresis (ECP), also known as extracorporeal photoimmunotherapy or photochemotherapy, is a leukapheresis-based therapy which was initially used in patients with cutaneous T-cell lymphoma (CTCL) (1). Specifically for the treatment of therapy refractory CTCL patients suffering from the leukemic variant, the Sézary Syndrome, ECP received FDA (United States Food and Drug Administration) approval in 1988. During ECP, whole blood of the patient is collected via a cubital vein, or a permanently implanted catheter, for separation of leucocytes from plasma and non-nucleated cells. With a specifically constructed device for this procedure, collected leukocytes, the so called buffy coat, are then exposed to ultraviolet-A (UVA) irradiation in the presence of a photosensitizing agent, 8-methoxypsoralen prior to reinfusion to the patient (Figure 1). Two basically different methods for performing ECP procedure have been described. They differ in the device used for leukocyte collection and UVA irradiation: the “closed system” and the so called “open system.” The closed system is based on the original design by Edelson and coworkers and is the only FDA-approved system. The open system is a system incorporating different separation instruments, mostly used outside the United States. No prospective comparative studies have been performed. Although ECP is a valid treatment method since 30 years and over 2 million of treatments have been performed, there are no reports about negative cytogenetic effects. Petersheim et al. investigated the mitotic index (MI), type and number of chromosomal aberrations after ECP treatment and could demonstrate that ECP is not associated with an increased mutagenic risk (2).

Over the last decades, indications for initiating ECP were continuously extended since its introduction. ECP treatments are generally well-tolerated by patients and there are almost no significant unwanted side effects. Taken together, ECP combines an excellent safety profile with efficacy. The aim of this article is to (1) introduce this technology, (2) give an overview where ECP has been showing promising effects and has become an essential part of treatment methods, (3) and to give recommendations on how to proceed in multiple indications.

## Mode of action

It has been 35 years since the first study on ECP was completed and 30 years since ECP was approved by the United States Food and Drug Administration (FDA). Nonetheless, the mode of action is still vaguely known, although many achievements have been made over the last decades. Research has shifted from mainly exploring new indications for ECP to a better understanding of the mechanisms of action in order to extend again the use of ECP for a wider range of diseases, but now with a clearer focus in mind (3).

Early studies ascribed the therapeutic effect of ECP to the initiation of apoptosis in lymphoid cells (4, 5). For this purpose, the photosensitizer 8-MOP was combined with exposure to UVA (320–400 nm), a concept which originally derived from the use of oral psoralen plus UVA (PUVA)-therapy but with the important difference that instead of 8-MOP-photosensitized skin (conventional oral PUVA therapy), buffy coat incubated with 8-MOP was exposed to UVA (ECP). UVA irradiation of cells after incubation with 8-MOP leads to DNA crosslinking. After reinfusion, subsequent apoptosis of lymphoid cells, largely natural killer (NK) cells and T-cells, arises (6).

While these proposed mechanisms might explain the therapeutic effect of ECP on CTCL, it does not elucidate how ECP should work in other indications. Hence, researchers' view on possible mechanisms of action shifted to a merely immunomodulatory approach. In line, a recently published consensus of the American Council of ECP underlines the importance of dendritic antigen-presenting cells (DCs) in the mechanisms of action of ECP (3).

Activation of monocytes occurs after contact with extracorporeal surfaces, which can be found in the tubing and the radiation chamber of the ECP device. Activated monocytes differentiate to immature DCs (iDCs) and consecutively get loaded with patient-specific antigens. These cells show characteristic surface markers of iDCs (CD83, X-11, Alpha-V, Beta-V, CD1a) (3, 7–10). The mechanism promoting differentiation to iDCs seems to relate to direct UVA effects and/or exposure of the buffy coat to extracorporeal surfaces (11). Upon reinfusion, phagocytosis of lymphoid cells is performed by iDCs, which subsequently undergo maturation and present antigenic peptides. This process has been named transimmunization (12).

It has been observed that the cytokine composition in the peripheral blood (increase of TNF-alpha and IL-6) changes after reinfusion of 8-MOP and UVA treated cells into the patient (13). An increase of CD36+ macrophages, due to the changes in tumor necrosis factor (TNF)-alpha and interleukin (IL)-6 levels, can be found after ECP. Hence, an immune response shift occurs which normalizes the imbalance of the Th1/Th2 response that can be found in CTCL. Summarizing, anti-inflammatory cytokines may be induced by ECP, whereas pro-inflammatory cytokines may be reduced (14, 15). As this may be beneficial for CTCL, the effect in autoimmune diseases must follow a different pathway. Indeed, in patients with graft-vs.-host disease (GVHD), ECP shifts the cytokine profile toward a Th2 immune response. Comparing the cytokine profiles before and after ECP in these patients, an increase of IL-4, IL-10 and transforming growth factor (TGF)-beta and a decrease of IL-12, IL-1, interferon-alpha, and TNF-alpha was observed, resulting in the apoptosis of mononuclear cells (16, 17).

Activation of T-cells leads to a differentiation into several cell lineages, particularly regulatory T-cells (Tregs) playing an important role in the down-regulation of immune reactions. Especially in patients with acute GVHD (aGVHD), Treg differentiation after ECP is highly reinforced and a significantly higher number of Tregs is noticeable in the peripheral blood in GVHD patients after ECP (18, 19). In a murine model 8-MOP and UVA-treatment induced Tregs similar to UVB-induced antigen specific Tregs characterized by the expression of CD4, CD25, CTLA-4, and Foxp3. In addition, it has been demonstrated that IL-10 is involved in this process (20–22). ECP might highly efficiently stimulate Tregs as has been shown in a murine model by Gatza et al. (18), Rezvani et al. (23), Zhai et al. (24), and Wolf (25). In the area of solid organ transplantation, ECP has been gaining more and more acceptance. In lung transplanted patients, a slight up regulation of CD4+CD25+Foxp3+ Tregs has been reported, possibly contributing to an increased immunotolerance of transplanted tissues and organs and hence survival rates (26).

In summary, research shifted from apoptosis induced by exposure to psoralen with UVA to an immunomodulatory approach, which is based on the initiation of dendritic cells, a modification of the cytokine profile and the stimulation of several T-cell lineages, in particular regulatory T-cells. Nonetheless, different pathways contribute to the beneficial effects of ECP in different indications and the final role of regulatory T cells has yet to be definitively established.

## Indications

### Cutaneous T-cell lymphoma (CTCL)

Cutaneous T-cell lymphoma (CTCL) represents a lymphoproliferative disorder primarily characterized by skin involvement due to accumulation of malignant T-cells. The most common subtypes of CTCL are mycosis fungoides (MF) and Sézary Syndrome (SS), which account for more than half of all CTCL patients. MF often resembles eczema or psoriasis in an initial phase, but is characterized by a clonal T-cell population. Patients often suffer from itchy plaques, but with disease progression nodular lesions and tumors may appear. In SS atypical mononuclear cells with a cerebriform nucleus (Sézary cells) appear which can be found in the skin, peripheral blood and lymph nodes. SS usually has a bad prognosis with a 5-year survival rate of 24% (27, 28). Initial treatment of CTCL is directed at the cutaneous involvement to improve quality of life and minimize the risk of reoccurrence. With disease progression, the addition of immune modulatory treatments, chemotherapy or stem cell transplantation may become a necessity (28, 29).

The first investigational study using ECP was performed in patients with the leukemic variant (Sézary Syndrome) of CTCL. In a meta-analysis for the efficacy of ECP, a response rate of 55.7% and a complete remission rate of 17.6% could be reported (1). A better response rate was noticed in patients with a low count of Sézary cells and low CD4/CD8 ratio. Patients with a low number of CD4+CD7-cells may also have a higher benefit from ECP. A combination of ECP with immune modulatory treatment may enhance the benefit of ECP (28, 30, 31). With the leukemic variant of CTCL as the oldest indication for ECP, many studies support the first-line use of ECP. A combination therapy can also be performed, with optimal response being attributed to the combination of ECP, interferon-alpha and bexarotene (31).

ECP has been established as a first-line treatment in CTCL patients with blood involvement (stage IVA1 or IVA2) and erythrodermic stage IIIA or IIIB (30, 32, 33). Treatment recommendations stated 2-weekly cycles of treatment on 2 consecutive days for at least 3 months and subsequent treatment every 3–4 weeks. Re-evaluation of treatment response should be performed between months 6 and twelve. If response is seen, treatment should be continued every 4–8 weeks. Combination of therapies can be considered, if ECP fails as first-line treatment (31, 34).

### Graft-Vs.-host disease (GVHD)

Although allogeneic hematopoietic stem cell transplantation (HSCT) is a potentially curative treatment of hematologic diseases, GVHD is still a limiting factor for the outcome of these patients (35). With possible involvement of multiple organs such as the skin representing the most common appearance, GVHD in liver, gut and in rare cases in lung and neuromuscular system are reported. According to the Consensus of National Institute of Health further sub-classification can be done into acute and chronic GVHD (36, 37). Corticosteroids remain first-line therapy for both acute and chronic GVHD but due to its association with significant toxicity and an increasing number of patients developing steroid-refractory disease, many salvage therapies are currently available. Based on recently published literature, mammalian target of rapamycin (mTor)-inhibitors (Sirolimus), janus kinase (JAK)-inhibitors (Ruxolitinib), proteasome inhibitors (Bortezomib), and also interleukin (IL)-22 are showing promising efficacy in the treatment of GVHD (38). For the treatment of chronic GVHD, Ibrutinib, an irreversible inhibitor of Bruton's tyrosine kinase (BTK), and Interleukin-2 inducible T-cell kinase (ITK), was recently granted FDA approval and is currently the only one approved for this purpose (39).

ECP is a widely recommended treatment modality as a second-line treatment, particularly in steroid-refractory form of GVHD. Current recommendations indicate that treatment should be performed on 2 consecutive days every week or every 2 weeks until a response is noticeable. ECP Treatments should be continued for at least 8 cycles or until complete remission is occurring (40). In a retrospective multicenter analysis, ECP has shown response rates of 80% in acute and chronic GVHD patients (41). A meta-analysis reviewed 7 prospective studies on acute GVHD and found overall good response rates but also a necessity of further prospective controlled multicenter studies (42). In a recently published article, the use of ECP as an initial prophylactic treatment was discussed, indicating its beneficial effect (43). An uncontrolled, prospective trial was able to show promising results for prophylactic use which has still to be confirmed in future studies (44).

### Scleroderma

Scleroderma is an autoimmune connective tissue disease characterized by increased fibroblast activation leading to hypertrophic dermal collagen. Skin involvement is just one appearance, beside joints and internal organs. Scleroderma is usually subdivided into a systemic (generalized) and a more localized form Zhou and Choi (45) and Gabrielli et al. (46). The pathogenesis of scleroderma is not well understood, however, Th2 and Th17 cells with accompanied cytokines, together with changes in number and function of Tregs might be related to the development of scleroderma (45, 47–49). Current treatment is based on immunosuppression, which include topical and systemic steroids, azathioprine, cyclophosphamide, methotrexate, mycophenolate mofetil (MMF), or interferons. Phototherapy is also a major component in the treatment of scleroderma and ranges from narrowband to broadband UVB, UVA, UVA1, PUVA, and ECP (50).

The use of ECP for scleroderma has been investigated in single patients with refractory disease (51, 52). A few larger treatment series are available. Treatment regime was usually performed on 2 consecutive days with a re-treatment every 2–6 weeks with a follow-up of usually 12 months. The effect of ECP was also investigated in randomized, double blind, placebo controlled studies with varying outcome, ranging from no improvement against no treatment, improvement over no treatment but no improvement against sham to a superiority of ECP against D-penicillamine treatment (53–60). Patients with scleroderma may have a higher risk in developing lung cancer, but no difference was found between patients with ECP and patients without ECP treatment (61).

Concluding the results of the published studies, best evidence of the use of ECP in scleroderma is given for skin manifestations, although joint involvement may also benefit. Scleroderma is an indication for ECP with a category III (grade 2B) by the American Society of Apheresis. This is supported by other guidelines which identify ECP as a second-line or alternative treatment in refractory patients (34, 62).

### Solid organ transplantation

Based on recently published statistical data from Eurotransplant, ~5,500 transplantations of solid organs were performed in 2017, with an ever continuously increasing number (63). Although major improvements in surgical techniques and new immunosuppressive protocols have been made, the long-time survival of transplanted patients is still limited due to acute and chronic allograft rejection, as well as opportunistic infections.

The first investigational study using ECP in the field of solid organ transplantation was performed in cardiac transplant rejection in 1992. By assessing endomyocardial biopsies after ECP treatments, successful reversal of acute cardial rejection could be observed (64, 65). Further studies in heart transplant recipients suffering from acute or chronic rejection were able to prove efficiency of ECP in reducing frequency and degree of rejection severity, without higher incidence of infections (66–69). In one study a significant reduction of cardiac allograft vasculopathy (CAV) in the ECP group determined by intravascular ultrasound was demonstrated (70).

Similar results by initiating ECP in the lung transplantation setting could be documented. Several trials presented efficient clinical response in the treatment of chronic rejection. Benden et al. examined the use of ECP in patients with bronchiolitis obliterans syndrome (BOS) and recurrent acute rejection after lung transplantation and were able to demonstrate that ECP reduced the rate of decline in lung function in BOS patients. In addition, patients suffering from recurrent acute rejection were clinically stabilized (71). Jaksch et al. were able to confirm the clinical improvements in BOS patients showing stabilization of lung function and significant greater survival (72). Greer et al. performed a retrospective analysis of all patients treated with ECP for chronic allograft dysfunction demonstrating stabilization as well as improvement in forced expiratory volume in 1 s (FEV1) (73). A recently published meta-analysis emphasizes the beneficial effect of ECP for clinical improvement of BOS (74). Nonetheless prospective, randomized controlled studies with a larger cohort are still missing to validate these results.

Several trials have been performed using ECP in the treatment of acute and chronic rejection after solid organ transplantation, though there is only one study examining the effect of ECP in prophylactic use. Cardiac transplant recipients were randomized to receive standard triple immunosuppressive therapy or additionally ECP treatments within the first month of transplantation. Promising results could be detected in the prevention of chronic rejection by decreased levels of non-donorspecific panel reactive antibodies (PRA) and decreased coronary artery intimal thickness in the ECP treated group (70). Data on using ECP as prophylaxis for allograft rejection in lung transplantation recipients is still missing and currently a highly relevant topic.

Recommendations are well established for patients suffering of BOS after lung transplantation and ECP treatment should start as soon BOS is diagnosed. In heart transplantation, ECP can be considered as an additional treatment. Cycles should be performed on 2 consecutive days with one cycle every 2 weeks for 3 months. After this initial phase, treatment intervals can be prolonged to once every month. It is still unclear how long ECP treatment should be continued, with ranges of 6–24 cycles. Continued treatment may be helpful in good responding patients with an improvement of clinical function (i.e., FEV1 in lung transplantation) (34).

### Crohn's disease

Crohn's disease (CD) represents an inflammatory condition, which can affect the entire gastrointestinal tract. This topographic distinction is often used to separate CD from ulcerative colitis, which mainly affects the colon, although the terminal ileum and colon are also primary affected by CD. Complications of the disease range from stricturing to penetrating complications after chronic inflammation. Intestinal surgery is often initiated after serious complications (75). The disease arises from hyperimmunity and chronic inflammation of the mucosa (76). It is therefore reasonable, that immunosuppression, such as steroids, methotrexate, TNF-alpha blockers, and other agents are a major component in the treatment of the disease. When using monotherapy or combined immunosuppression, the risk of infections are usually a limitation and restrict treatment success (77).

The use of ECP in CD is still not well established. In a pilot study with treatment on 2 consecutive days every 2 weeks for 12 cycles, a withdrawal from steroid therapy in almost half of the ECP treated patients could be reached, without relapsing symptoms. In almost all other patients, steroid dose could be reduced by at least half of the initial dose (78). In uncontrolled prospective studies, ECP was well tolerated and clinical response was initiated in half of the patients with a remission rate up to 25% and a significant reduction of steroid doses (79, 80). The use in pediatric patients is an unexplored area, but a case report is in accordance to the results seen in adults (81).

### Atopic dermatitis

Atopic dermatitis (AD), also known as atopic eczema, is a chronic relapsing skin disease, mainly characterized by itchy skin lesions. Severity is often represented by the affected area of the skin (82–84). Skin lesions of AD are histologically characterized by epidermal changes. These include spongiosis and epidermal hyperplasia, combined with dermal infiltrates consisting of T-lymphocytes, monocytes, and eosinophilic cells. A genetic background is often involved in this multifactorial disease (85). On a cellular level, a malfunction of Tregs and an impaired Th2/Th17-driven immune response to antigens can be observed, that leads to skin changes (86, 87). Standard therapy for adults usually includes topical steroids, calcineurin inhibitors, or phototherapy (i.e., UVA-1, PUVA, or UVB). In refractory cases, systemic therapy becomes a necessity. Promising results have been achieved using the IL-4 receptor antagonist dupilumab, which has been approved by the EMU/FDA in 2017 (88, 89). In selected severe, otherwise refractory cases, the use of rituximab or intravenous IgG (IVIG) might be an option.

The use of ECP for AD has already been performed for almost 25 years with the first publication in 1994 by Prinz et al. (90). After these initial three patients with good response, several open label studies were conducted that proof usefulness of ECP in standard therapy refractory AD patients with a significant decrease of affected skin area (91–99). Although the clinical effect of ECP in AD is limited, patients with refractory disease might benefit from ECP in combination with topical or systemic treatment.

### Type 1 diabetes

Type 1 diabetes (T1D) is a T-cell mediated autoimmune disease where T-cells are directed against pancreatic insulin-producing beta-cells. Management of this disease is usually performed with blood glucose control self-monitoring and insulin injections. Severity can be graded on the remaining beta-cell function. The lower the remaining insulin production, the higher the risk of long-term complications (100, 101). Because beta-cell function is a vital predictor of disease severity, the preservation of these cells plays a crucial role in the management of this disease. Evidence shows that beta-cells have a regenerating ability (102). The exact autoimmune pathogenesis remains vague, but it is evident, that autoreactive CD4+ and CD8+ T-cells play an important role in the destruction of pancreatic beta-cells, whereas other autoantibodies may also be involved in this process (103). Summarizing the conditions in T1D, an imbalance of the immune system is occurring and the solitary suppression of the immune response does not seem adequate, considering the adverse events (104–106).

In a non-obese diabetic mouse model, cells treated with ECP were reinfused and the development of T1D was significantly delayed. An immune regulatory process is likely to occur in this scenario and Foxp3+ Tregs may be involved (107). Only one study is available, where ECP was used in newly diagnosed T1D patients. The group of children treated with ECP produced more C-peptide and needed significantly lower doses of insulin per kg bodyweight (82).

In conclusion, few studies are available for the evaluation of usage of ECP for T1D, but published data shows promising results as an additional therapy to delay the onset of T1D. Because ECP was well tolerated in the clinical trial, further studies on young patients may improve the outcome of this autoimmune disease.

## Conclusion

Since the first prospective trial on the use of ECP was performed by Edelson et al., multiple promising results in various entities have been published in the last decades. ECP found its establishment in the treatment of different diseases and acceptance as an immunomodulatory therapy with high potential of inducing tolerance. To date, no significant side effects have been reported. Due to its excellent safety profile, ECP is more and more investigated in prospective randomized trials with larger cohorts—on the one hand to extend its clinical indication with a clearer focus, and on the other hand to examine the complexity of the underlying immunomodulatory mechanism of action. Further research on identifying biomarkers which could predict the response to ECP is required.

## Author contributions

AC designed a concept, performed literature search, and wrote the manuscript. CJ gave additional ideas and performed correction of manuscript. RK performed supervision and final correction.

### Conflict of interest statement

The authors declare that the research was conducted in the absence of any commercial or financial relationships that could be construed as a potential conflict of interest.
